# Modeling Causal Relationships among Brain Areas in the Mesocorticolimbic System during Resting-State in Cocaine Users Utilizing a Graph Theoretic Approach

**DOI:** 10.4172/2329-6488.1000279

**Published:** 2017-08-31

**Authors:** Suchismita Ray, Bharat B Biswal, Ashley Aya, Suril Gohel, Aradhana Srinagesh, Catherine Hanson, Stephen J. Hanson

**Affiliations:** 1 Center of Alcohol Studies, Rutgers University, Piscataway, NJ 08854, USA; 2 Biomedical Engineering, New Jersey Institute of Technology, Newark, NJ 07102, USA; 3 School Of Health Related Professions-Biomedical Informatics, Rutgers University, Newark, NJ 07101, USA; 4 Rutgers University Brain Imaging Center, Newark, NJ 07102, USA

**Keywords:** Cocaine, Effective connectivity, Resting-state, Mesocorticolimbic system

## Abstract

**Objective:**

While effective connectivity (EC, causal interaction) between brain areas has been investigated in chronic users of cocaine as they view cocaine pictures cues, no study has examined EC while they take part in a resting-state scan. This resting-state fMRI study aims to investigate the causal interaction among brain areas in the mesocorticolimbic system (MCLS), which is involved in reward and motivation, in cocaine users (vs. controls).

**Method:**

Twenty cocaine users and 17 healthy controls finished a structural and a resting-state scan. Mean voxel-based time series data were obtained from brain regions of interest (ROIs) from the MCLS, and were input into a Bayesian search algorithm called IMaGES.

**Results:**

The causal interaction pattern was different between the two groups. The feed-forward pattern found in cocaine smokers, between 7 ROIs of the MCLS during resting-state [ventral tegmental area (VTA)→hippocampus (HIPP)→ventral striatum (VenStri)→orbital frontal cortex (OFC), medial frontal cortex (MFC), anterior cingulate cortex (ACC), dorsolateral prefrontal cortex (DLPFC)], was absent in controls. That is, the subcortical VenStri area had a causal influence on four cortical brain areas only in cocaine users.

**Conclusions:**

During the resting-state scan, the VTA of cocaine smokers abstinent for at least 72 hours, but not controls, begins causal connections to limbic, midbrain, and frontal regions in the MCLS in a feed-forward manner. Following replication, further studies may assess if changes over time in EC during resting-state predict cocaine treatment efficacy and outcome.

## Introduction

The mesocorticolimbic system (MCLS) has been linked to reward, motivation and goal-directed behavior. It contains the brain structures, namely, the ventral striatum (nucleus accumbens), extended amygdala, hippocampus, anterior cingulate, prefrontal cortex, and insula [1,2]. Drugs of abuse enhance extracellular dopamine concentration in these structures of the MCLS, which are triggered by dopaminergic projections mainly from the ventral tegmental area (VTA) [2]. According to the previous studies [3–5], although the MCLS reacts to natural rewards such as food, water, and sex, drugs of abuse induce a larger response in this system than physiological stimuli. Past research suggests that the drugs of abuse “hijack” the neurobiological mechanisms by which the brain reacts to reward, creates reward-related memories, and summarizes action repertoires leading to the reward [6,7].

Using functional magnetic resonance imaging (fMRI) technique, resting-state functional connectivity (RSFC; how individual brain regions are integrated during resting-state) has been examined on MCLS in cocaine users [8]. Also, a few effective connectivity (EC; the causal influence that one brain region exerts over another) studies have been conducted on cocaine users during resting-state scans and experimental tasks (working memory, response inhibition) [8]. The purpose of the present research was to investigate EC among all the MCLS brain areas [2] in cocaine smokers at resting-state using fMRI. This research is an extension of Gu et al. [9] and Ray et al. [8], and provides the first comprehensive examination of EC within cocaine users’ MCLS, utilizing baseline BOLD signal. The spatial patterns of resting-state correlation data are stable, in that they are similar across multiple resting-states (such as eyes-open and fixation) and across individuals and sessions. Because of the lack of task demands, resting-state design minimizes problems associated with experimental design and participant agreement, thus making it attractive to do research on clinical populations (“Components of the Human Connectome Project-Resting-state fMRI,” no date).

In this research, we investigated the causal interactions among all MCLS brain areas while participants’ brains were scanned at rest. This approach is valuable in terms of understanding how communication among MCLS brain areas changes due to neuroadaptation pertinent to long-term cocaine exposure. In the context of the cue-reactivity study, we collected resting-state fMRI data from non-treatment seeking cocaine users who were abstaining from cocaine for three days and similarly-aged controls. This research used IMaGES (Independent Multisample Greedy Equivalence Search; Ramsey et al., [10,11] to model EC during resting-state. IMaGES was specifically developed to reveal feed-forward effective networks based on functional time series data. Utilizing IMaGES allows us to understand how the subcortical areas causally influence cortical areas. IMaGES is a method of model detection rather than model fitting and can accommodate an essentially unlimited number of brain regions in any given model [11]. The algorithm works by exploring all possible graph solutions for the given set of ROIs rather than fitting the fMRI data to a specified model as is done with Granger causality or dynamic causal modeling (DCM) algorithms. IMaGES thus reduces prior assumptions about EC, implicit and explicit, associated with other graph analysis methods that require a priori specification of a network model to be fitted. The connectivity graphs produced by IMaGES have also been shown to be consistent with structural connections obtained using Diffusion Tensor Imaging (DTI) data [12].

We propose that as VTA projects to many regions in the MCLS, these connections allow for suitable comparison between cocaine smokers and control participants during resting-state. More specifically, we hypothesized that in cocaine users, but not in controls, VTA will connect to limbic (amygdala, hippocampus (HIPP)), midbrain (ventral striatum (VenStri)), and frontal areas (orbital frontal cortex (OFC), medial frontal cortex (MFC), anterior cingulate cortex (ACC), dorsolateral prefrontal cortex (DLPFC)) in a feed-forward manner [13] during resting-state.

## Methods

### Participants and procedure

In a research done by Ray et al., [8] each participant needed to complete a resting-state scan and a high resolution anatomical MPRAGE (magnetization-prepared rapid acquisition with gradient echo) scan. Participants were instructed during the resting-state scan to lie quietly without any movements and to visually fixate on a fixation cross for six minutes. All participants first completed the resting-state scan and then took part in a cue exposure task.

### Image acquisition

Imaging data were collected using a 3T Siemens Trio head-only fMRI scanner equipped with a standard Siemens head coil. While participants visually fixated on the cross, T2*-weighted echo planar images were acquired (35 axial slices, voxel size 3 × 3 × 3 mm, interslice gap 1 mm, matrix size 64 × 64 mm, FOV = 192 mm, TR = 2000 ms, TE 25 ms, flip angle = 90°) covering the entire brain. A sagittal T1-weighed structural scan (TR=1900 ms, TE = 2.52 ms, matrix = 256 × 256, FOV =256 mm, voxel size 1 × 1 × 1 mm, 176 1- mm slices with .5 mm gap) was acquired in order to co-register it with the fMRI data [14].

### ROI selection

The nine bilateral ROIs included: VTA, HIPP, VenStri, OFC, MFC, ACC, DLPFC, amygdala, and insula [2].

### Data Pre-processing

For imaging data pre-processing procedure. Time series data for each ROI for each participant in each group were fed to the IMaGES program. IMaGES produced two outputs: one for the cocaine smoking group and one for the controls. Unlike other EC measures, such as Granger Causality or DCM, IMaGES models directionality of the edges. IMaGES are based on regression, not correlation. A detailed explanation of the method and its approach to causality can be found in Ramsey et al (2010).

## Results

### Motion comparison

Unpaired t-test results showed that the mean frame-wise displacement was not significantly different between the cocaine and control groups (p =0.8120).

### IMaGES outputs

**Figure 1:**
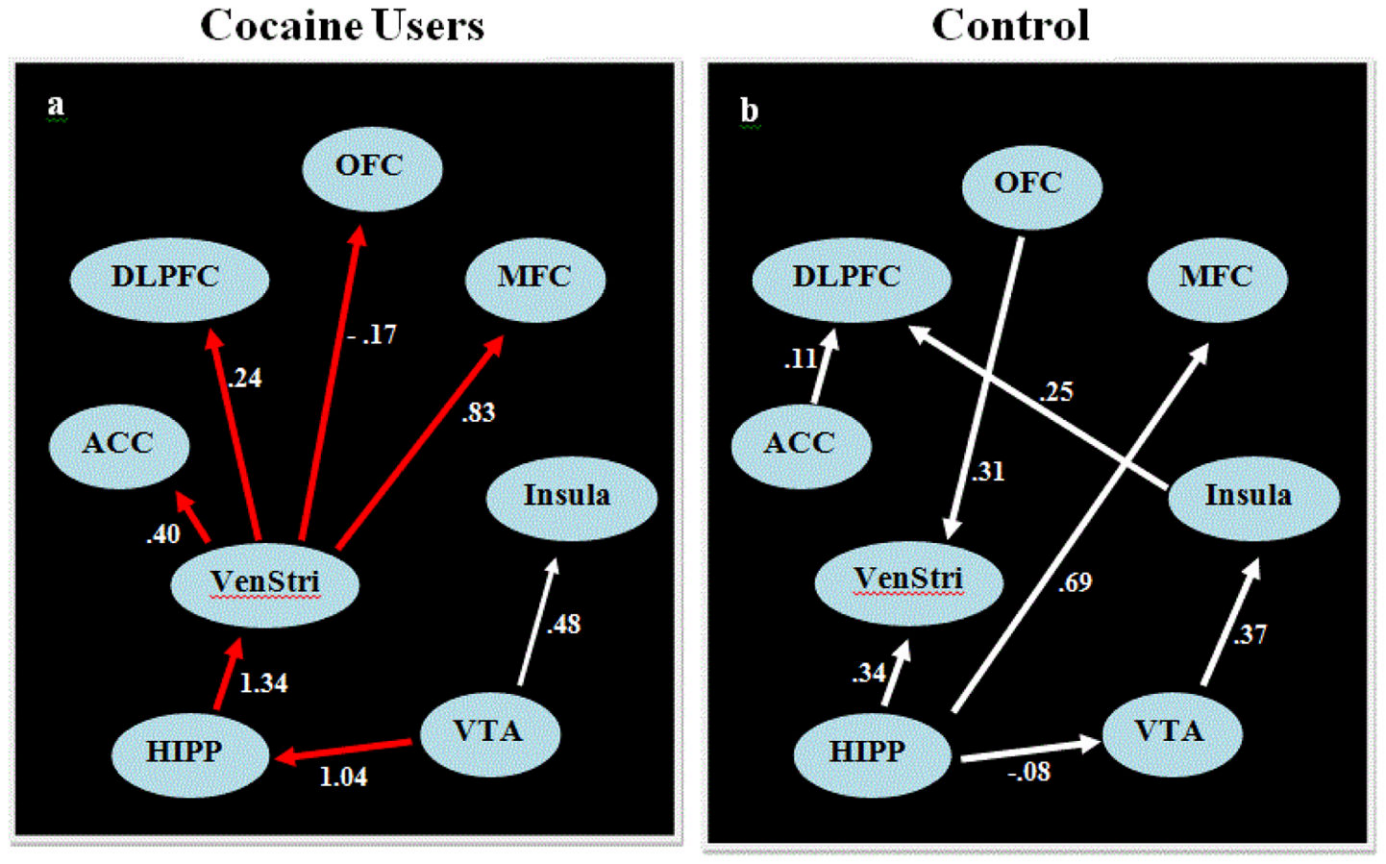
Comparison of the causal interaction between brain regions within the mesocorticolimbic system in cocaine users to healthy controls during resting-state. In Figure 1, the direction of arrows represents a direct causal influence of one region of interest (ROI) on another. The numbers on the arrows are regression coefficients and they denote the strength of causal influence of one ROI on another. The red arrows represent that in cocaine users, but not in controls, ventral tegmental area connected to limbic (hippocampus), midbrain (ventral striatum), and frontal areas in a feed-forward manner during resting-state (ventral tegmental area → hippocampus → ventral striatum → orbital frontal cortex, medial frontal cortex, anterior cingulate cortex, dorsolateral prefrontal cortex). Note: HIPP = hippocampus, VenStri = ventral striatum, ACC = anterior cingulate cortex, MFC = medial frontal cortex, DLPFC = dorsolateral prefrontal cortex, OFC = orbital frontal cortex, VTA = ventral tegmental area.

Figure 1 portrays resting-state data in cocaine users and control participants. The arrow direction shows a direct causal influence of one ROI on another. Numbers represent regression coefficients, which show the strength of causal influence. IMaGES could not produce any output using all nine ROIs as the time series of amygdala was linearly-dependent with that of multiple ROIs. Therefore, amygdala had to be excluded from the IMaGES analysis.

IMaGES showed that for smokers at resting-state (Figure 1a), the VTA started two feed-forward pathways. One feed-forward pathway included 7 ROIs. VTA causally influenced the HIPP, and then HIPP activated the VenStri, which then activated four frontal brain areas: the OFC, MFC, ACC, DLPFC (VTA→HIPP→VenStri→OFC, MFC, ACC, DLPFC). The other feed-forward pathway involved VTA causally activating insula.

In controls (Figure 1b), HIPP, but not VTA, initiated three feed-forward causal pathways. One feed-forward pathway involved 4 ROIs: HIPP directly activated the VTA, and the activation of the VTA then caused activation in insula, which then directly activated the DLPFC. There were two other feed-forward pathways that HIPP created: it directly caused activation in the VenStri and in the MFC.

Thus, the causal interaction pattern found between the two groups was different, providing evidence for different underlying processing models during resting-state [15,16]. The feed-forward connection involving 7 ROIs starting with VTA in cocaine users included limbic, midbrain, and frontal brain areas, which was absent in controls. Also, the subcortical VenStri area had a causal influence on four cortical brain areas only in cocaine users. In Figures 1a and 1b, any link that appears in the IMaGES graphs was significant, based on the Bayesian Information Criteria (BIC) for model selection.

## Discussion

Overall, the purpose of this analysis was to use IMaGES to compare EC among all MCLS brain areas in chronic users of cocaine to that of healthy controls during resting-state. In accordance with our hypothesis, for cocaine smokers the VTA was connected to limbic (HIPP), midbrain (VenStri), and frontal areas in a feed-forward manner during resting-state (VTA→ HIPP→VenStri→OFC, MFC, ACC, DLPFC). Differences in the IMaGES graphs in the cocaine group compared to the control group required different solutions, which supported the underlying model of proposed differences during resting-state [15,16].

The present study extends previous research by Ray and colleagues (2016) by establishing that cocaine smokers’ VTA initiates a feed-forward causal interaction between the 7 regions in the MCLS at resting-state. It is noteworthy that the present findings are consistent with Ray and colleagues (2016) in that the VTA showed a direct activation upon HIPP during resting-state. However, the spectral DCM model in Ray et al. (2016) could not reveal the feed-forward connections between VTA, limbic, midbrain, and frontal areas, as identified in the current IMaGES analysis. Also, the current results are similar to findings in the same cocaine individuals during the cocaine cue exposure task [1]. Subcortical areas causally influenced cortical areas. However, one unique connection that was present in cocaine smokers while they saw cocaine picture cues (insula → DLPFC) [1] was absent in the current resting-state findings. Possibly, this unique connection was a result of an increased craving in cocaine smokers versus controls while they were exposed to cocaine picture cues. The smokers demonstrated a significant positive correlation between the strength of causal influence of insula on DLPFC and the craving rating during cocaine picture cue exposure [1]. Interestingly, the present results showed that if the cocaine smokers’ onset of cocaine use was recent, they had a stronger causal influence of VenStri on DLPFC.

The direct activation of VTA on HIPP and on insula in chronic cocaine users is consistent with Jasinska et al. [2] who proposed that addictive drugs enhance dopaminergic projections mostly from the VTA to other MCLS regions. The present study expands previous RSFC studies involving drug users. That is, Wilcox et al. [17] demonstrated that cocaine users showed an increased RSFC between VenStri and OFC; and Ma et al. [18] observed that heroin users showed an enhanced RSFC between VenStri and ACC, and between VenStri and OFC. Importantly, this research further showed that during resting-state, VenStri causally influenced the frontal brain regions, for example, OFC and ACC in cocaine smokers.

In the present control participants, the HIPP initiated three feedforward causal pathways (Figure 1b: HIPP → VTA → insula → DLPFC; HIPP → VenStri; HIPP → MFC). We speculate that these EC pathways, specifically activation of DLPFC by both insula and ACC, and activation of MFC by HIPP, may reflect the activation of temporal memory in non-drug users [19]. DLPFC and MFC appear to play a role in temporal memory processes, providing a record of the order in which stimuli and events have occurred over time [19,20]. Thus, activation in these two brain areas in control participants might indicate their initiation of thoughts involving the events that have occurred over time in the recent past.

We speculate that activation of the HIPP in the initial stage of the MCLS in cocaine users may be related to long-term memories of their past drug use [21,22], whereas the subsequent activation of frontal brain areas may be related to processes involving prolonged drug use [2].

This study also had a few limitations. First, even though the cocaine and control groups were equated for age, education, and ethnicity, controls had increased alcohol intake in comparison to cocaine smokers. However, alcohol use was modest in both groups (mean 1 drink/day) and therefore unlikely to have affected the results. Second, since we only had five female smokers, possible sex differences in EC during rest could not be investigated. Third, since amygdala ROI time series signal was correlated with the time series of multiple ROIs, IMaGES could not produce output using all nine ROIs. Amygdala had to be excluded from the IMaGES analyses. Even with these constraints, this research serves as a model of EC among MCLS ROIs at rest in chronic cocaine smokers.

## Conclusion

This research is the first demonstration in chronic cocaine smokers (but not in controls) that VTA initiates causal connections to limbic, midbrain, and frontal brain regions in MCLS in a feed-forward manner during resting-state scan. If these results are replicated, future research may assess if changes over time in EC during resting-state predict cocaine treatment efficacy and outcome.
